# Comparison of three different lipid removal cleanup techniques prior to the analysis of sulfonamide drug residues in porcine tissues

**DOI:** 10.1002/fsn3.1158

**Published:** 2019-08-07

**Authors:** Jie Wang, Qiuhui Hu, Peng Li, Yong Fang, Wenjian Yang, Ning Ma, Fei Pei

**Affiliations:** ^1^ College of Food Science and Engineering Nanjing University of Finance and Economics Nanjing China

**Keywords:** enhanced matrix removal for lipid, hydrophilic‐lipophilic balance, mass spectrometry, porcine tissues, sulfonamides

## Abstract

A number of 17 sulfonamides (SNs) determination in porcine tissues using two new materials including Enhanced Matrix Removal for Lipid (EMR‐L) and Oasis PRiME hydrophilic‐lipophilic balance (HLB), and the conventional liquid–liquid extraction with n‐hexane (LLE) sample preparation methods were evaluated and compared. Samples were extracted uniformly with acidified acetonitrile and cleaned up by the three sample preparation methods, and then, analytes were further separated by ultrahigh‐performance liquid chromatography (UHPLC) and detected by a triple quadrupole mass spectrometry (MS/MS) or a quadrupole‐time‐of‐flight tandem mass spectrometry (Q‐TOF/MS). The results showed that the matrix effects from the EMR‐L and HLB were significantly lower than that from LLE, suggesting that these two new materials are superior to n‐hexane in the precipitation of proteins and the adsorption of lipids. Moreover, the recoveries of 17 SNs were quantified by the matrix‐matched calibration curve at spiked level of 5, 10, and 20 μg/kg, and 97.0% of the results satisfied method validation requirements. The samples cleaned up by EMR‐L and HLB achieved the highest average recovery in liver and kidney with high moisture content, and muscle which is high in fat, respectively. In addition, Q‐TOF/MS could play a good role in aided verification based on the result of repeated validation test. In summary, either combination of approaches could be used to achieve monitoring purposes; it is still worthwhile to adopt a specific sample preparation method and MS detector for the quantification in a specific matrix.

## 1 INTRODUCTION

Veterinary drugs have been widely used in livestock husbandry as feed additive to promote growth rate, treat diseases, and provide high‐quality products for profitability in the market. Sulfonamides (SNs), as synthetic antibacterial veterinary drugs, have been widely used in clinics. It has a broad‐spectrum antibacterial property with chemical stability and ease of use (Kawai et al., 2016; Zessel, Mohring, Hamscher, Kietzmann, & Stahl, 2014). Moreover, the combined use of SNs and other drugs, such as trimethoprim (TMP), can synergistically enhance the antibacterial effect and expand the treatment range (Economou, Petraki, Tsipi, & Botitsi, 2012; Li, Sun, Zhang, & Pang, 2013). However, overuse of SNs can lead to excessive residues in animal‐derived foods, which may endanger human health and cause environmental pollution (Graham et al., 2011; Oberlé, Capdeville, Berthe, Budzinski, & Petit, 2012). Currently, more than half of the world's pork is consumed in China, and such a huge demand presents challenge for ensuring the quality and safety of the pork products and supervising the slaughter process (Xiong, Liu, Chen, & Zheng, 2017; Yu & Abler, 2014). Therefore, an effective and systematical method for SNs detection in pork products is needed.

Animal‐derived foods are possessed of complex matrix, as well as numerous interfering substances, and are not easily extracted and cleaned up. Traditional sample preparation methods for SNs in food include liquid–liquid extraction (LLE; Adesiji, Deekshit, & Karunasagar, 2014; Biselli, Schwalb, Meyer, & Hartig, 2013), solid‐phase extraction (SPE) (Stolker et al., 2008), and the quick, easy, cheap, effective, rugged, and safe (QuEChERS) approach (Costa, Caldas, & Primel, 2014). Every extraction method has its own advantage, for instance, LLE functions achieve the purpose of extraction and cleanup mainly through a variety of analytes using the mutual insolubility of two different solvents (Li et al., 2019; Zhan et al., 2012). SPE is a method of purifying the extraction based on the physical and chemical properties of each analyte to select the appropriate materials and extraction conditions (Peters, Bolck, Rutgers, Stolker, & Nielen, 2009). Acetonitrile (MeCN) or acidified acetonitrile was served as extractant, C_18_, primary secondary amine (PSA), graphene were typically used as adsorbents for QuEChERS, which is commonly applied to sample preparation of veterinary drugs with enhanced analytical efficiency (Rúbies, Antkowiak, Granados, Companyó, & Centrich, 2015; Wang & Leung, 2012; Zhang et al., 2016). Despite many previous studies, there are still some claimed shortcomings. For example, LLE requires the use of excessive organic reagents, SPE blocks column easily, and QuEChERS has less selectivity for some substances (Garrido Frenich, Aguileraluiz, Martínez Vidal, & Romerogonzález, 2010; Wang et al., 2011; Yoshikawa et al., 2017). Aiming at these problems, a new product from Agilent Technologies called “Enhanced Matrix Removal for Lipid” (EMR‐L) could selectively capture the long hydrocarbon chain of lipids (Han, Matarrita, Sapozhnikova, & Lehotay, 2016). Cleanup ability of QuEChERS and protein precipitation extracts were well integrated by EMR‐L due to the activated material (López‐Blanco et al., 2016). Meanwhile, Oasis PRiME HLB (HLB) is a popular representative reverse‐phase SPE adsorbent developed by Waters, which is commonly used in the cleanup of trace substances. HLB does not require activated and balanced because of special solid‐phase packing (Chen et al., 2016; Han et al., 2015). Currently, EMR‐L and HLB have been widely used in the detection of veterinary drug residues in animal‐derived foods (Zhao, Lucas, Long, Richter, & Stevens, 2018). Nevertheless, to the best of our knowledge, no reports have been available on the profiles for the comparison of performances of EMR‐L and HLB methods.

Ultrahigh‐performance liquid chromatography coupled to triple quadrupole mass spectrometry (UHPLC‐MS/MS) technology provides a broad‐based method for the widest range of veterinary drugs and dominates in the field of veterinary drug analysis (Clark, Storey, & Turnipseed, 2011). It can simultaneously identify and quantify hundreds of targeted analytes to meet the daily monitoring needs of food contaminants (Park et al., 2015). Meanwhile, high‐resolution MS (HRMS) instruments such as quadrupole‐time‐of‐flight tandem mass spectrometers (Q‐TOF/MS) provide accurate mass measurements, defined as the ability to determine the mass of a compound within a few parts‐per‐million (ppm) of its theoretical mass (Turnipseed, Lohne, & Boison, 2015). The applicability of Q‐TOF/MS for the determination of veterinary drugs in animal food products at or below the levels required by regulatory agencies has been demonstrated (Rodriguez‐Cabo, Rodriguez, Ramil, & Cela, 2016).

The aim of this study was to systematically evaluate the analytical and practical performance of three sample preparation methods (EMR‐L, HLB, and LLE) by UHPLC‐MS/MS in the analysis of 17 SNs spiked into extracts of porcine tissues (muscle, liver, and kidney) at regulatory levels. The feasibility of reducing false‐positive results by Q‐TOF/MS had also been validated.

## 2 MATERIALS AND METHODS

### 2.1 Chemicals and reagents

MeCN, methanol, n‐hexane, and formic acid were supplied by Merck. Ultrapure water was prepared with a Milli‐Q system by Millipore. All solvents were in chromatographic grade.

Analytical standards (≥98%) of sulfadiazine (SD), sulfadimethoxine (SDM), sulfamerazine (SM), sulfamethazine (SM2), sulfamethizole (SMT), sulfamethoxazole (SMZ), sulfathiazole (STZ) sulfachlorpyridazine (SCD), trimethoprim (TMP), sulfamethoxypyridazine (SMD), sulfapyridine (SPD), sulfadoxine (SDX), sulfisoxazole (SIZ), sulfabenzamide (SBA), sulfaquinoxaline (SQX), sulfacetamide (SAA), and sulfaphenazole (SPZ) were purchased from Sigma‐Aldrich. Stock solutions were prepared at a concentration of 100 µg/ml in methanol, and mixed standard solutions were diluted to 1,000 ng/ml. All solutions were stored at −20°C.

Oasis PRiME HLB (6cc, 200 mg) was purchased from Waters. EMR‐L product was purchased from Agilent Technologies, which consisted of two 15‐ml centrifuge tubes. QuEChERS d‐SPE EMR‐L contained 1 g EMR‐L material, and QuEChERS Final Polish EMR‐L contained 1.6 g magnesium sulfate and 0.4 g sodium chloride for salting out.

### 2.2 Sample preparation

In a comparison of the three methods, 2 g of samples (*n* = 6 for each) from muscle, liver, and kidney were minced and homogenized before weighed into 50‐ml centrifuge tubes spiked with SNs and extracted with 10 ml MeCN containing 0.1% formic acid. The samples were vortex‐mixed for 5 min at room temperature using a pulsating vortex platform shaker (Anting). Then, the sample tubes were placed in an ultrasonic bath (Xinzhi) at 70°C for 10 min to extract the veterinary drugs more fully out of the matrix through the ultrasonic‐assisted extraction. Extracts were centrifuged at 2,862 rcf for 10 min, and the obtained upper layer was decanted into a new centrifuge tube for the further cleanup as follows.

#### 2.2.1 Enhanced matrix removal for lipid method

Ultrapure water (5 ml) was added to the QuEChERS d‐SPE EMR‐L tube and vortex‐mixed immediately for 15 s, in order to activate the EMR‐L material. The upper layer (5 ml) was transferred into the tube and vortex‐mixed for 5 min and ultrasonically cleaned for 10 min, then centrifuged at 2,862 rcf for 10 min.

In the salting‐out step, 5 ml of upper layer was transferred into the QuEChERS Final Polish EMR‐L tube. These sample tubes were vortexed, ultrasonically cleaned, and centrifuged as described above. Thereafter, 5 ml of upper layer was pipetted into 15‐ml graduated glass centrifuge tubes evaporated to near dryness at 40°C under N_2_ flow, and then reconstituted to 1 ml by MeCN.

#### 2.2.2 Hydrophilic‐lipophilic balance method

The upper layer (5 ml) was loaded into the Oasis PRiME HLB tube under gravity and the last few drops of extractant gently pushed through with a pipet bulb. Extracts were evaporated to dryness and reconstituted as described above.

#### 2.2.3 Liquid–liquid extraction method

MeCN‐saturated n‐Hexane (10 ml) was added into the polypropylene centrifuge tube that contained the upper layer and vortex‐mixed immediately for 30 s. Then, the upper layer n‐hexane was transferred, and the above steps were repeated once to clean up the lipid. The final subnatant was evaporated to dryness and reconstituted in the same step.

### 2.3 UHPLC‐MS/MS analysis

A Waters Acquity ultrahigh‐performance liquid chromatography system (Ireland) coupled to a Xevo TQ‐S triple quadrupole mass spectrometer. Chromatographic separations were developed in an Infinity Lab Poroshell 120 EC‐C18 column (2.1× 100 mm, 2.7 μm), acquired from Agilent Technologies. The mobile phases consisted of (A) MeCN and (B) water, each containing 0.1% formic acid. The flow rate was 0.2 ml/min, and the column oven temperature was maintained at 40°C. The injection volume was 5 μl, and the initial gradient was 5% A. The gradient was as follows: 0–2.5 min, 5% A‐55% A; 2.5–5 min, 55% A‐90% A; and 5–7 min, 90% A‐5% A.

The data station operating software was MassLynx version 4.1. For the MS/MS detection, instrument was operated in ESI + mode using the scheduled multiple reaction monitoring (MRM) mode. The ionization source parameters were as follows: capillary voltage, 2.3 kV; desolvation temperature, 450°C; desolvation gas (N_2_) flow rate, 850 L/min; and cone gas (N_2_) flow rate, 50 L/min. The values of retention time, collision energy, and cone voltage of all veterinary drugs in the MRM mode are given in Table 1.

**Table 1:** Retention time (TR) and other UHPLC‐MS/MS conditions (CE, collision energy; CV, cone voltage) for SNs

Drug	TR (min)	Precursor ion (m/z)	Production ion (m/z)	CE (V)	CV (V)	Ion ratio (%)
SD (Sulfadiazine)	2.80	251.04	92^a^, 156	27, 15	30	86
SDM (Sulfadimethoxine)	3.43	311.12	92, 156^a^	32, 20	36	78
SM (Sulfamerazine)	3.01	265.11	92^a^, 156	25, 15	35	89
SM2 (Sulfamethazine)	3.16	279.27	156, 186^a^	18, 16	15	81
SMT (Sulfamethizole)	3.13	271.13	92, 156^a^	25, 15	30	94
SMZ (Sulfamethoxazole)	3.37	254.12	92^a^, 156	25, 15	30	99
STZ (Sulfathiazole)	2.91	256.11	92^a^, 156	25, 14	30	88
SCD (Sulfachlorpyridazine)	3.25	285.15	92^a^, 156	28, 15	32	75
TMP (Trimethoprim)	2.91	291.32	123^a^, 230	30, 30	40	98
SMD (Sulfamethoxypyridazine)	3.12	281.21	92^a^, 156	30, 16	20	82
SPD (Sulfapyridine)	2.93	250.17	108^a^, 156	25, 16	33	96
SDX (Sulfadoxine)	3.36	311.18	92^a^, 156	32, 15	35	97
SIZ (Sulfisoxazole)	3.31	268.11	92^a^, 156	28, 13	30	88
SBA (Sulfabenzamide)	3.47	277.19	92^a^, 156	25, 15	30	80
SQX (Sulfaquinoxaline)	3.42	301.13	92, 156^a^	30, 16	32	99
SAA (Sulfacetamide)	2.61	215.01	108, 156^a^	18, 12	25	94
SPZ (Sulfaphenazole)	3.43	315.26	158^a^, 160	35, 30	20	83

a Transitions for quantification.

### 2.4 Q‐TOF/MS analysis

A Shimadzu Nexera UHPLC system was coupled to an AB Sciex 5,600^+^ Triple TOF mass spectrometer. Conditions for chromatographic separation was the same as the program used in UHPLC‐MS/MS.

The data station operating software was Analyst 1.7. The Q‐TOF/MS instrument was operated in ESI+ mode using scheduled information‐dependent acquisition (IDA) mode. The TOF‐MS method was tuned for the 100–1000 mass range under the following conditions: accumulation time, 0.1 s; ion source gas 1 (N_2_), 55 μl/min; ion source gas 2 (N_2_), 55 μl/min; curtain gas (N_2_), 35 μl/min; source temperature, 550°C; ion spray voltage floating, 5,500 V; declustering potential, 80 V; and collision energy, 10 V. The product ion (+) IDA method was the same as above except that the accumulation time was 0.05 s, the collision energy was 35 V and the collision energy spread was 15 V.

### 2.5 Quantification and matrix effects

The recoveries were calculated by comparing spiked samples with matrix‐matched (MM) calibration standards. Standard solution (SS) and MM calibration curves were fortified at concentrations of 1.0, 5.0, 10.0, 20.0, and 50.0 μg/kg for each sample preparation method (Anumol, Lehotay, Stevens, & Zweigenbaum, 2017).

To calculate matrix effects, the slope of the SS calibration curves was compared with the slope of the MM calibration curves (ME = k_MM_/k_SS_ − 1). The matrix led to enhancement effects when the value was positive, whereas the matrix led to suppression when the value was negative. When the values ranged from −20% to +20%, matrix enhancement or suppression effects were considered acceptable.

## 3 RESULTS AND DISCUSSION

### 3.1 Comparison of extractives

**Figure 1:**
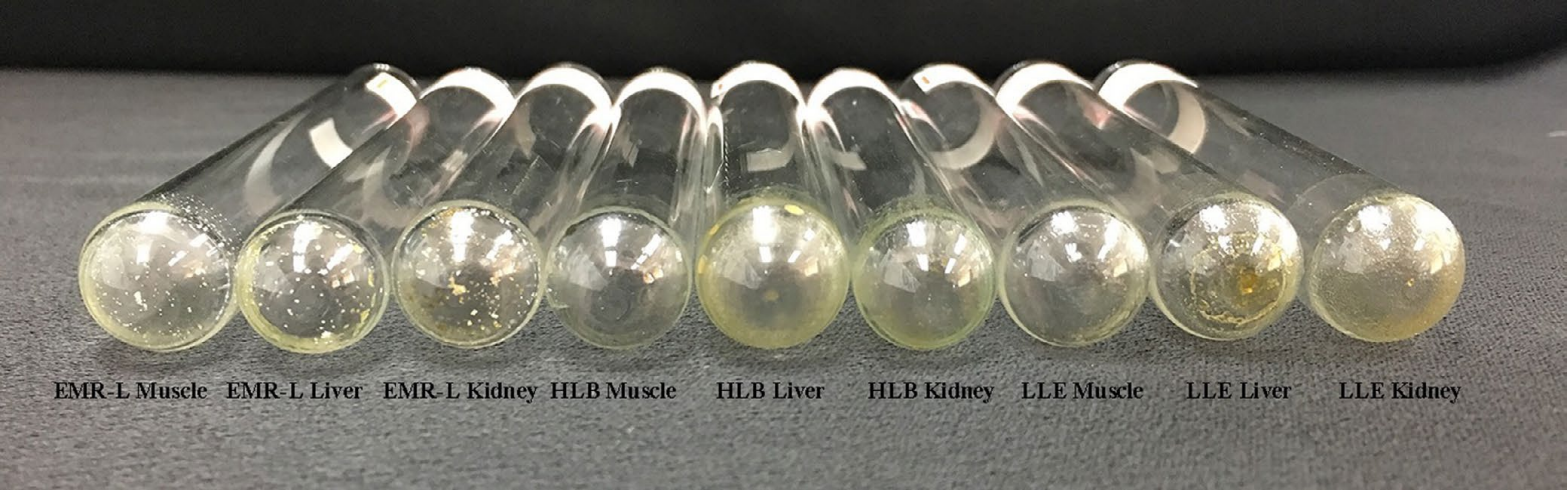
Extractives from porcine tissues with three sample preparation methods

Comparison of extractives is an important way to evaluate the feasibility of sample preparation method for high‐throughput screening. Extractives (1 ml) were, respectively, dropped into a glass tube and dried in the oven at 80°C, and the extractives of muscle, liver, and kidney were processed by the three sample preparation methods (Figure 1). According to the cleanliness of the bottom of the glass test tube, the EMR‐L and HLB gave cleaner matrix extractives than LLE method, due to their specific material. The EMR‐L tubes consisted of magnesium sulfate, special polymeric materials, and sodium chloride. The salts in the EMR‐L extractives were brought in by salting‐out step (Kaufmann, Butcher, Maden, Walker, & Widmer, 2014). Moreover, HLB could effectively adsorb lipids due to the polymerized solid‐phase packing of lipophilic divinylbenzene and hydrophilic N‐Vinylpyrrolidone (Li et al., 2017). Therefore, samples with higher moisture content such as liver and kidney cleaned up by EMR‐L gave the same cleanliness as HLB‐cleaned up muscle.

### 3.2 Matrix effects

Matrix effects are common in LC‐MS analysis of complex samples, especially in ESI^+^ mode (Dasenaki & Thomaidis, 2015). Appropriate acidity can promote the mass spectrometer ionization efficiency of ESI^+^ (Gómez Pérez, Romero‐González, Martínez Vidal, & Garrido, 2014), and MeCN is a suitable solvent for extracting SNs and has the function of precipitating proteins to improvement of the ionization efficiency of the target analyte. In order to facilitate the quantification of this experiment, the method of using acidified MeCN extraction followed by cleanup with different adsorbents was adopted. Matrix‐matched calibration, which was widely used to reduce matrix effects in practice, was performed in this study. Matrix effects for all compounds which were detected by UHPLC‐MS/MS are presented in Table 2. It can be seen that matrix effects for most of analytes prepared using three different methods rarely exceed ±20%, and most analytes produced a slight ionization suppression. However, SD and SIT for LLE method were subjected to significant signal suppression in muscle and liver, respectively. The matrix effects of LLE were more severe than the EMR‐L and HLB cleanup methods. For a sample individual, the matrix effects of muscle, liver, and kidney are not regular. For example, Tiele, Maiara, Osmar, Martha, and Renato (2016) used the LLE method to detect veterinary drug residues in liver and kidney, and the matrix effect of liver samples was higher than that of kidneys. Granelli and Branzell (2007) developed an LLE method for detecting antibiotics in muscle and kidney. For some antibiotics, the matrix effect in muscle was higher than in the kidney.

**Table 2:** Matrix effects for SNs tested in porcine tissues for three sample preparation methods by UHPLC‐MS/MS

Drug	Matrix effects (%)								
	EMR‐L			HLB			LLE		
	Muscle	Liver	Kidney	Muscle	Liver	Kidney	Muscle	Liver	Kidney
SD	−6.78	−0.66	−2.63	−4.56	−3.38	−3.86	−23.87	0.18	−3.66
SDM	0.21	−0.34	0.13	0.16	−0.34	0.34	−5.58	0.21	0.31
SM	−0.54	−0.49	−0.81	0.09	−0.51	0.16	−5.66	−1.95	−1.72
SM2	−0.29	−0.31	−0.02	−0.52	0.12	−0.19	−2.34	−0.42	−0.04
SMT	−0.28	0.44	0.04	0.21	−0.01	0.51	−18.53	−0.09	0.42
SMZ	−5.33	−1.55	−2.21	−8.44	−0.21	−0.34	−10.65	0.19	−6.94
STZ	−6.39	−2.09	−0.48	−5.41	0.03	−1.12	−16.4	−0.32	−3.28
SCD	−0.09	−0.16	−0.59	0.03	−0.57	0.44	−1.08	−0.72	0.21
TMP	−0.23	0.58	−0.24	−0.21	0.43	−2.49	−0.56	−1.87	−2.81
SMD	−2.31	−0.07	−3.45	−0.16	0.56	−0.31	−12.06	−7.98	−4.55
SP	−0.88	2.13	−0.23	−0.72	5.13	11.19	−3.07	−11.27	−8.31
SDX	−1.05	0.07	0.29	−0.22	−0.29	−4.91	−1.78	−1.13	0.87
SIZ	0.05	−1.04	10.21	−0.58	−8.61	5.56	−3.45	−25.18	4.63
SBA	0.11	0.92	0.59	−0.46	−0.03	0.82	−2.54	0.72	0.85
SQX	−0.13	0.52	0.18	−0.22	−0.24	0.35	−2.24	0.56	0.46
SAA	0.96	−5.21	−3.17	−0.67	2.46	−1.72	−1.52	−2.48	0.71
SPZ	0.49	−4.86	2.07	0.11	−2.28	3.88	−3.18	0.61	3.84

### 3.3 Linearity, LODs, LOQs, Recoveries, and RSDs

MM calibration was used to better quantitate these analytes when evaluating sample preparation methods in this study (Valese et al., 2017). LODs and LOQs were determined by separately considering a signal‐to‐noise (S/N) ratio equal to 3 and 10. The linear range of the 17 SNs was 1.0–50.0 μg/kg. LODs (LOQs) ranged from 0.04 to 0.23 μg/kg (0.13–0.56 μg/kg), 0.08 to 0.41 μg/kg (0.27–1.36 μg/kg), and 0.21 to 1.07 μg/kg (0.70–3.57 μg/kg) for EMR‐L, HLB, and LLE, respectively. Compared with other literatures, the LODs/LOQs values are lower (Deng et al., 2011; Xie et al., 2012). This means that these three methods for detecting SNs residues meet industry standards. Which indicate that the 17 SNs in porcine tissues cleaned up by the three methods have a good linearity in the range of 1.0–50.0 μg/kg.

The extracts were analyzed with the UHPLC‐MS/MS method, and recoveries were calculated by MM calibration curves in the range of 5.0–20.0 μg/kg, each in sextuplicate. Average recoveries of the analytes mostly range from 70% to 120% in three matrices (Table 3). The average recoveries of tested drug have small differences among the muscle (82.44%, 92.50%, and 79.85%, for EMR‐L, HLB, and LLE, respectively), liver (95.09%, 91.12%, and 82.86%, for EMR‐L, HLB, and LLE, respectively), and kidney (85.11%, 82.71%, and 79.71%, for EMR‐L, HLB, and LLE, respectively). EMR‐L and LLE present lower average recovery in the cleanup of muscle. At the same time, HLB shows the best results for samples with the large amount of lipids and proteins. Meanwhile, EMR‐L‐cleaned up shows better recovery than the other in liver and kidney, just as it appeared in the extractives. This can be due to its better efficiency with high moisture content samples. It is also worth noting that almost all of the three sample preparation methods reveal low recovery in the kidney, which might be caused by the presence of uncleared metal ions (Lehotay et al., 2012). In any case, the overall recovery meets the methodological validation, showing the feasibility of porcine matrix analyzed by UHPLC‐MS/MS.

**Table 3:** Recovery and RSD for SNs spiked at 5, 10, and 20 μg/kg tested in porcine for three sample preparation methods by UHPLC‐MS/MS, *n* = 6 replicates

Drug	Spiked Level (μg/ kg)	%Recovery. (%RSD.)								
		EMR‐L			HLB			LLE		
		Muscle	Liver	Kidney	Muscle	Liver	Kidney	Muscle	Liver	Kidney
SD	5	84.44 (5.15)	104.50 (3.43)	84.21 (2.97)	92.81 (3.77)	93.13 (3.63)	79.29 (2.67)	81.59 (6.41)	80.13 (18.03)	68.39 (3.27)
	10	81.38 (0.44)	79.59 (3.65)	77.03 (12.03)	95.52 (3.45)	91.22 (0.52)	73.59 (4.93)	81.84 ((4.97)	95.17 (6.97)	83.01 (2.82)
	20	86.62 (4.11)	102.76 (6.93)	85.78 (5.28)	97.25 (3.33)	101.33 (1.84)	77.72 (1.91)	83.61 (5.13)	86.29 (8.82)	75.43 (9.47)
SDM	5	81.47 (5.78)	94.74 (5.52)	75.46 (5.30)	96.09 (4.07)	97.19 (7.13)	70.78 (1.93)	74.83 (8.22)	74.42 (7.34)	74.27 (4.62)
	10	78.46 (1.32)	79.71 (8.13)	80.41 (7.33)	80.07 (3.53)	86.45 (5.62)	78.94 (6.84)	82.09 (4.61)	86.14 (5.69)	81.69 (3.67)
	20	78.64 (0.81)	115.12 (5.28)	84.28 (11.07)	107.46 (3.51)	97.67(3.29)	87.52 (4.92)	81.14 (5.87)	83.58 (9.36)	61.52 (3.22)
SM	5	78.43 (3.13)	108.37 (1.88)	85.46 (2.36)	109.01 (4.79)	94.73 (4.22)	72.37 (1.87)	76.63 (8.22)	75.62 (15.78)	79.12 (0.77)
	10	78.09 (2.94)	73.46 (4.17)	81.79 (9.01)	82.77 (6.87)	92.77 (4.01)	81.33 (3.09)	86.94 (3.27)	90.01 (8.16)	84.17 (4.96)
	20	80.31 (8.69)	116.04 (4.59)	91.47 (11.06)	83.76 (3.03)	100.89 (3.13)	85.48 (5.32)	83.52 (2.09)	85.83 (11.87)	93.39 (5.67)
SM2	5	82.81 (4.61)	99.37 (6.36)	90.38 (7.78)	81.91 (5.17)	84.98 (16.81)	73.84 (2.31)	75.18 (4.23)	71.69 (15.53)	80.22 (1.13)
	10	78.34 (1.86)	89.76 (10.48)	108.38 (13.76)	100.26 (3.29)	94.86 (4.62)	79.96 (6.07)	85.49 (5.87)	83.74 (6.28)	85.07 (3.77)
	20	80.27 (10.54)	126.19 (4.71)	84.02 (9.25)	82.14 (2.82)	97.91 (5.78)	73.98 (4.99)	73.14 (7.72)	86.31 (11.11)	77.92 (0.92)
SMT	5	87.61 (2.41)	101.63 (3.16)	80.14 (0.82)	89.49 (7.31)	93.63 (5.16)	81.12 (4.56)	79.06 (1.17)	77.02 (12.86)	75.90 ((0.92)
	10	82.13 (3.53)	83.71 (5.38)	96.89 (10.49)	89.81 (4.43)	93.98 (1.34)	76.68 (1.51)	88.87 (2.12)	87.28 (6.74)	79.77 (1.48)
	20	85.02 (1.72)	106.18 (6.83)	99.42 (7.01)	98.76 (4.36)	102.56 (3.16)	83.94 (5.77)	83.92 (6.24)	82.06 (6.57)	78.56 (1.47)
SMZ	5	85.77 (2.03)	104.54 (5.71)	84.12 (10.27)	91.99 (3.67)	73.61 (7.95)	81.36 (3.43)	76.68 (2.12)	71.11 (16.63)	74.09 (3.39)
	10	83.41 (4.16)	83.92 (2.36)	85.33 (8.82)	87.41 (4.52)	77.88 (2.04)	76.40 (5.38)	86.82 (2.67)	86.22 (6.32)	80.22 (1.63)
	20	85.42 (3.07)	103.72 (4.99)	86.18 (10.33)	90.13 (4.47)	93.16 (3.93)	84.19 (2.53)	80.49 (3.57)	82.49 (11.17)	89.93 (3.74)
STZ	5	82.24 (4.19)	100.94 (3.93)	86.15 (8.41)	86.28 (3.13)	85.47 (4.87)	96.37 (1.79)	75.42 (4.62)	73.05 (11.33)	75.69 (6.32)
	10	83.26 (5.56)	75.35 (7.81)	85.16 (10.56)	91.81 (2.07)	84.69 (2.22)	98.92 (3.62)	61.88 (2.07)	89.53 (9.59)	80.10 (2.47)
	20	83.37 (6.52)	98.16 (5.19)	83.34 (11.64)	99.86 (2.73)	98.97 (5.27)	97.79 (3.22)	81.03 (8.72)	85.58 (9.18)	82.44 (2.42)
SCD	5	77.56 (5.54)	76.59 (3.03)	80.86 (4.65)	88.67 (4.29)	80.38 (3.32)	97.24 (6.52)	80.54 (5.14)	90.83 (4.64)	73.59 (3.87)
	10	68.62 (2.62)	70.15 (5.88)	87.78 (7.39)	91.85 (2.17)	78.98 (3.19)	99.07 (5.18)	84.29 (0.92)	87.32 (10.03)	76.19 (3.22)
	20	74.41 (4.71)	81.07 (2.27)	91.51 (8.81)	106.51 (2.66)	92.99 (4.58)	80.71 (1.58)	84.21 (3.99)	86.48 (5.92)	82.62 (2.78)
TMP	5	76.24 (3.44)	95.08 (3.58)	82.43 (7.80)	99.95 (5.52)	87.92 (4.73)	75.86 (1.62)	76.33 (5.32)	76.44 (11.03)	75.54 (3.72)
	10	77.09(1.29)	75.73 (9.77)	78.94 (11.28)	81.94 (3.44)	84.63 (0.31)	84.07 (2.87)	78.97 (8.41)	84.93 (6.72)	90.82 (3.48)
	20	78.36 (4.71)	112.37 (4.89)	86.48 (9.55)	80.83 (9.19)	101.37 (4.36)	89.59 (4.91)	84.76 (5.67)	86.38 (12.46)	87.83 (4.41)
SMD	5	85.13 (4.63)	107.06 (7.19)	81.61 (14.37)	108.11 (4.18)	80.99 (6.84)	79.64 (2.67)	75.32 (1.72)	77.01 (16.25)	76.47 (2.72)
	10	80.21 (4.44)	77.78 (5.07)	80.08 (9.41)	90.32 (1.02)	93.32 (1.30)	77.23 (6.18)	88.20 (1.17)	88.23 (9.81)	75.82 (7.04)
	20	83.49 (2.49)	108.73 (4.58)	88.16 (11.26)	85.39 (2.83)	96.07 (2.14)	86.87 (6.29)	81.07 (2.43)	84.48 (11.98)	93.41 (0.66)
SP	5	83.74 (3.81)	99.71 (2.86)	106.93 (3.14)	91.85 (1.84)	87.66 (5.56)	72.66 (4.96)	76.09 (4.48)	73.87 (11.23)	84.87 (1.78)
	10	81.62 (1.67)	79.23 (4.87)	84.29 (12.49)	95.07 (4.01)	87.50 (2.22)	73.14 (5.94)	73.53 (3.56)	86.93 (6.79)	79.93 (2.76)
	20	84.54 (4.74)	107.88 (2.72)	84.37 (9.23)	98.26 (3.64)	96.89 (4.77)	82.43 (2.87)	70.27 (5.23)	90.57 (8.11)	86.58 (3.58)
SDX	5	82.06 (4.76)	106.06 (1.61)	87.18 (3.11)	94.07 (3.13)	91.97 (6.36)	80.65 (2.76)	75.39 (3.44)	76.13 (10.63)	67.07 (2.53)
	10	83.77 (0.24)	84.11 (5.07)	81.18 (12.38)	94.75 (5.34)	95.43 (1.72)	79.21 (3.62)	82.52 (1.61)	86.75 (7.78)	78.62 (3.18)
	20	85.14 (2.99)	104.38 (3.41)	87.11 (10.98)	95.94 (2.67)	93.44 (3.99)	82.72 (2.87)	77.54 (4.62)	82.12 (8.03)	80.21 (2.73)
SIZ	5	81.79 (0.21)	101.76 (7.26)	83.96 (4.77)	87.18 (6.63)	92.53 (7.27)	74.73 (7.66)	76.70 (5.88)	72.52 (10.09)	74.33 (5.70)
	10	82.28 (0.62)	79.09 (3.09)	77.43 (9.19)	92.63 (5.45)	93.42 (2.69)	78.19 (5.61)	67.39 (3.96)	83.40 (5.22)	79.68 (2.07)
	20	81.73 (4.11)	112.07 (4.53)	86.57 (8.46)	96.41 (5.79)	96.37 (5.17)	79.64 (2.22)	83.14 (5.18)	82.67 (7.95)	84.92 (2.33)
SBA	5	86.01 (6.09)	92.48 (6.51)	73.39 (1.58)	87.12 (0.61)	89.03 (5.33)	89.66 (1.39)	77.93 (2.61)	66.34 (11.42)	72.01 (1.22)
	10	82.17 (3.25)	75.88 (5.69)	74.13 (13.31)	91.37 (8.93)	93.34 (2.02)	97.84 (4.17)	82.97 (4.03)	89.43 ((3.91)	78.89 (5.64)
	20	82.42 (3.01)	107.61 (4.20)	84.12 (6.06)	99.52 (7.22)	95.97 (2.17)	83.67 (3.96)	82.16 (2.25)	82.58 (10.03)	84.91 (2.23)
SQX	5	96.27 (3.76)	98.53 (5.58)	82.29 (16.88)	96.99 (1.61)	76.59 (2.92)	82.61 (1.55)	84.74 (1.57)	66.75 (9.59)	87.09 (3.74)
	10	94.26 (4.65)	83.59 (1.95)	85.24 (12.23)	91.74 (6.77)	73.06 (0.39)	93.90 (1.92)	90.01 (2.39)	94.53 (5.15)	85.57 (12.63)
	20	89.14 (2.24)	103.76 (0.47)	87.12 (11.01)	84.56 (4.30)	91.67 (4.44)	93.93 (3.24)	70.62 (4.32)	82.27 (10.91)	92.43 (3.26)
SAA	5	83.32 (7.86)	94.01 (5.71)	80.83 (5.83)	94.57 (4.34)	89.16 (10.13)	89.25 (4.06)	83.09 (3.97)	75.82 (11.03)	69.57 (4.88)
	10	80.01 (3.07)	79.57 (3.06)	92.89 (7.91)	83.12 (2.59)	96.05 (4.83)	88.56 (4.12)	74.52 (7.93)	89.93 (4.25)	79.15 (3.62)
	20	85.36 (4.85)	103.66 (2.89)	84.14 (5.30)	91.07 (10.56)	90.43 (8.07)	85.64 (3.37)	85.37 (6.82)	88.84 (8.23)	81.86 (6.87)
SPZ	5	84.49 (5.19)	104.59 (3.43)	84.22 (2.99)	92.89 (3.75)	93.17 (3.69)	79.26 (2.62)	81.53 (6.44)	80.12 (18.01)	68.37 (3.21)
	10	81.34 (0.41)	79.57 (3.61)	77.01 (12.05)	95.50 (3.41)	91.23 (0.55)	73.59 (4.96)	81.88 (4.99)	95.13 (6.91)	83.01 (2.82)
	20	86.65 (4.11)	102.78 (6.97)	85.70 (5.21)	97.28 (3.38)	101.38 (1.82)	77.71 (1.94)	83.62 (5.15)	86.21 (8.84)	75.42 (9.47)

### 3.4 Repeated validation test by Q‐TOF/MS

**Figure 2:**
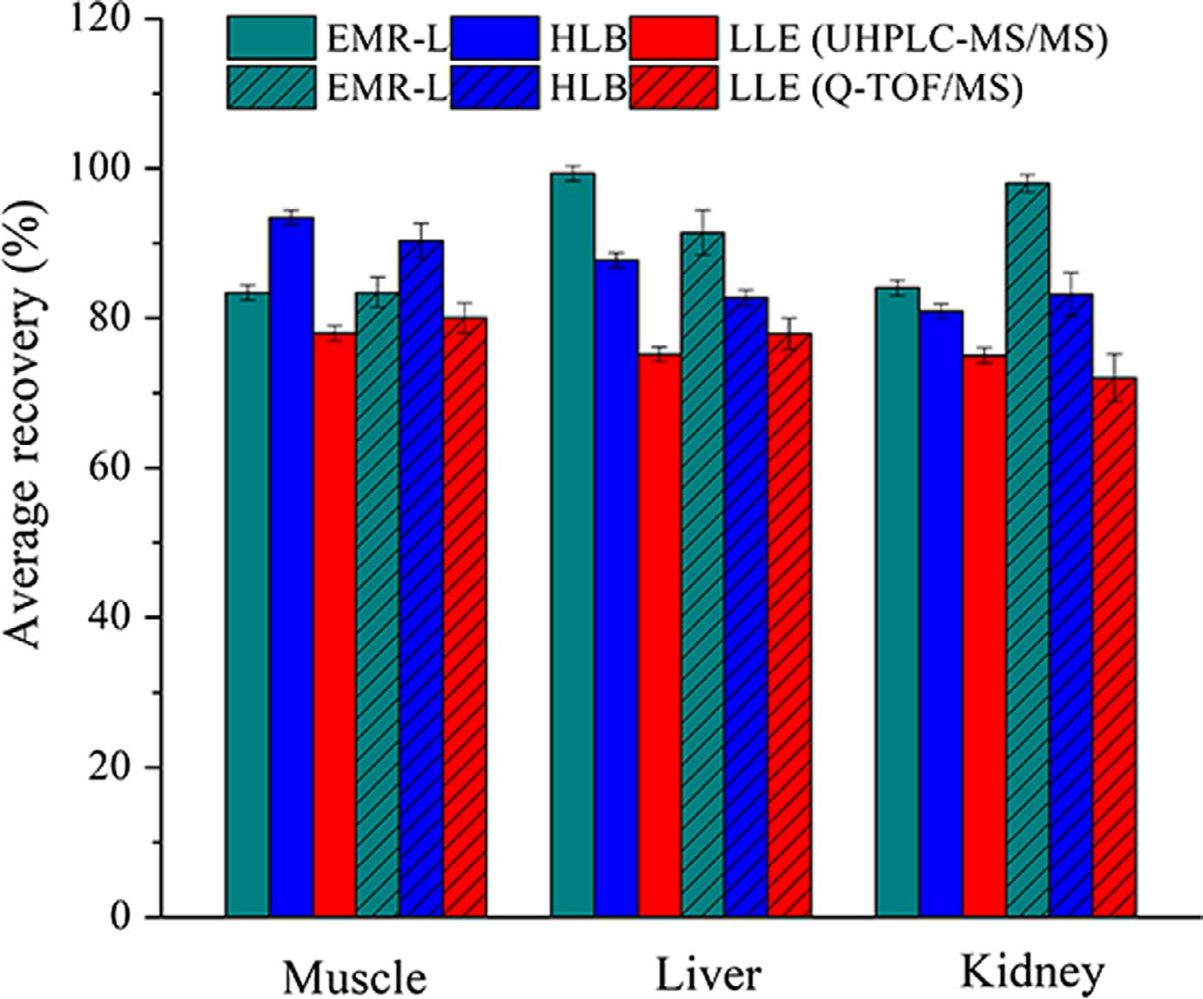
Average recovery of 17 SNs (5 μg/kg, *n* = 6) tested in three matrices with three sample preparation methods by UHPLC‐MS/MS and Q‐TOF/MS

The mass of ions could be accurately determined by using the Q‐TOF/MS, which is helpful for rapid screening. As a broad‐spectrum detection technology, Q‐TOF/MS provides an excellent qualitative ability to reduce the occurrence of false‐positive results. However, high qualitative ability usually comes with the risk of instability in quantitative ability, the use of accurate mass and full scan alone to detect analytes could lead to deviation in quantification (Wang, Li, Chang, Kang, & Pang, 2017). For aided verification analysis and elimination of false‐positive results, the above analytes were repeatedly injected into the Q‐TOF/MS. The average recoveries results (5 μg/kg, *n* = 6) are shown in Figure 2. The trend detected by Q‐TOF/MS was similar with UHPLC‐MS/MS, although the exact numbers of different sample preparation methods differ greatly. LODs (LOQs) ranged from 0.87 to 4.06 μg/kg (2.91–13.53 μg/kg), 0.93 to 3.71 μg/kg (3.1–12.37 μg/kg), and 1.72 to 4.96 μg/kg (5.73–16.53 μg/kg) for EMR‐L, HLB, and LLE, respectively. In addition, the detection limit of Q‐TOF/MS in the laboratory is 1.0 μg/kg due to the lack of sensitivity. That value meets the residue limit of SNs in pork according to EU Council Regulation 470/2009/EC (Council Regulation (EEC) (2009)) and the U.S. Food and Drug Administration in CFR Title 21 (United States Food & Drug Administration, 2015). Hence, Q‐TOF/MS is capable of performing high‐throughput screening for daily monitoring of those compounds with higher residue limits (Turnipseed, Storey, Clark, & Miller, 2011).

### 3.5 Comparison with the national standard method of China

In order to further verify the reliability of the method, the researched methods in this study were compared with the national standard method of China. UHPLC‐MS/MS was used to detect a positive muscle sample containing SMD. The content of sample is 16.3 ± 0.49 μg/kg (EMR‐L), 16.8 ± 0.35 μg/kg (HLB), 15.4 ± 0.91 μg/kg (LLE), and 16.6 ± 0.27 μg/kg (Chinese standard), respectively. The results of EMR‐L and HLB are similar to Chinese standard. In addition, both methods provide fewer sample preparation steps, thus saving time and labor costs.

## 4 CONCLUSIONS

In this study, the analytical and practical performance of three sample preparation methods (EMR‐L, HLB, and LLE) and two analytical methods (UHPLC‐MS/MS and Q‐TOF/MS) in the analysis of 17 SNs spiked into extracts of porcine tissues (muscle, liver, and kidney) were systematically evaluated. According to our results, EMR‐L and HLB were recommended to pretreat high moisture content or lipid matrix, respectively, as the result shown in matrix effects. The overall recovery met the methodological validation. LLE showed lower recovery and higher matrix effects in the SNs analysis compared with the other sample preparation methods, although it could be used as a rapid screening. In addition, Q‐TOF/MS results were confirmed that it can play a good role in aided verification based on the result of repeated validation test.

This study, therefore, demonstrates the feasibility of application of commercial product, HLB and EMR‐L, compared with the traditional LLE technology.

## CONFLICT OF INTEREST

No conflict of interest declared.

## ETHICAL APPROVAL

This study does not involve any human or animal testing.
